# Coexistence of primary central nervous system lymphoma and primary breast lymphoma: Clinical presentation, imaging features, and treatment management

**DOI:** 10.1016/j.radcr.2022.04.005

**Published:** 2022-05-09

**Authors:** Francesca Di Giuliano, Tommaso Perretta, Francesca Pitocchi, Noemi Pucci, Maria Lina Serio, Aurelia Caliandro, Eliseo Picchi, Valentina Ferrazzoli, Chiara Adriana Pistolese, Francesco Garaci, Roberto Floris

**Affiliations:** a Neuroradiology Unit, Department of Biomedicine and Prevention, University of Rome Tor Vergata, Viale Oxford 81, Rome, 00133, Italy; b Department of Biomedicine and Prevention, University of Rome Tor Vergata, Viale Oxford 81, Rome, 00133, Italy

**Keywords:** Primary central nervous system lymphoma, Primary breast lymphoma, Integrated imaging, Magnetic resonance, Mammography, PET/CT, Ultrasound

## Abstract

The presence of synchronous dual hematological diseases is an uncommon finding. We report an unusual case of coexistence of primary central nervous system lymphoma and primary breast lymphoma without systemic involvement in an immunocompetent patient. To our knowledge a similar case has not yet been reported in the literature. We especially focus on presenting the imaging features, the associated clinical findings and treatment management of each entity, with the aim of raising awareness on these two rare types of lymphomas and the possibility of their coexistence.

## ABBREVIATION

PBL: Primary Breast Lymphoma
PCNSL: Primary Central Nervous System
NHL: Non-Hodgkin Lymphoma
DLCBL: Diffuse Large Cell B Lymphoma
EBV: Epstein-Barr Virus
MRI: Magnetic Resonance Imaging
ADC: Apparent Diffusion Coefficient
DWI: Diffusion-Weighted Imaging
DCE: Dynamic Contrast-Enhanced
PET/CT: Positron Emission Tomography
FDG: Fluorodeoxyglucose
CSF: Cerebrospinal Fluid

## Introduction

Primary central nervous system lymphoma (PCNSL) and primary breast lymphoma (PBL) are 2 distinct extremely rare types of extra nodal non-Hodgkin lymphoma (NHL).

PCNSL is an aggressive form of NHL that occurs in the brain, spinal cord, leptomeninges, or eyes, without evidence of systemic involvement, it accounts for only 2%-3% of NHL cases and about 2%-4% of all primary brain tumors [1,2]. Diffuse large B-cell lymphoma (DLBCL) is the most common histological subtype of primary CNS lymphomas [3,4].

PBL accounts for less than 1% of all NHL, for <3% of extra nodal lymphomas and approximately 0.5% of breast malignancies [5], [6], [7], [8], [9], [10]. According to Wiseman and Liao criteria, modified by Hugh et al, PBL is defined by the presence of a primary lesion within the breast, having mammary tissues, and lymphomatous infiltrates in close proximity to each other, with no history of previous lymphoma or evidence of systemic disease and no extramammary sites of involvement other than ipsilateral axillary nodes [11,12].

We present a case of coexistence of DLBCL-PCNSL and T-cell-PBL in an immunocompetent patient.

## Case report

**Fig. 1:**
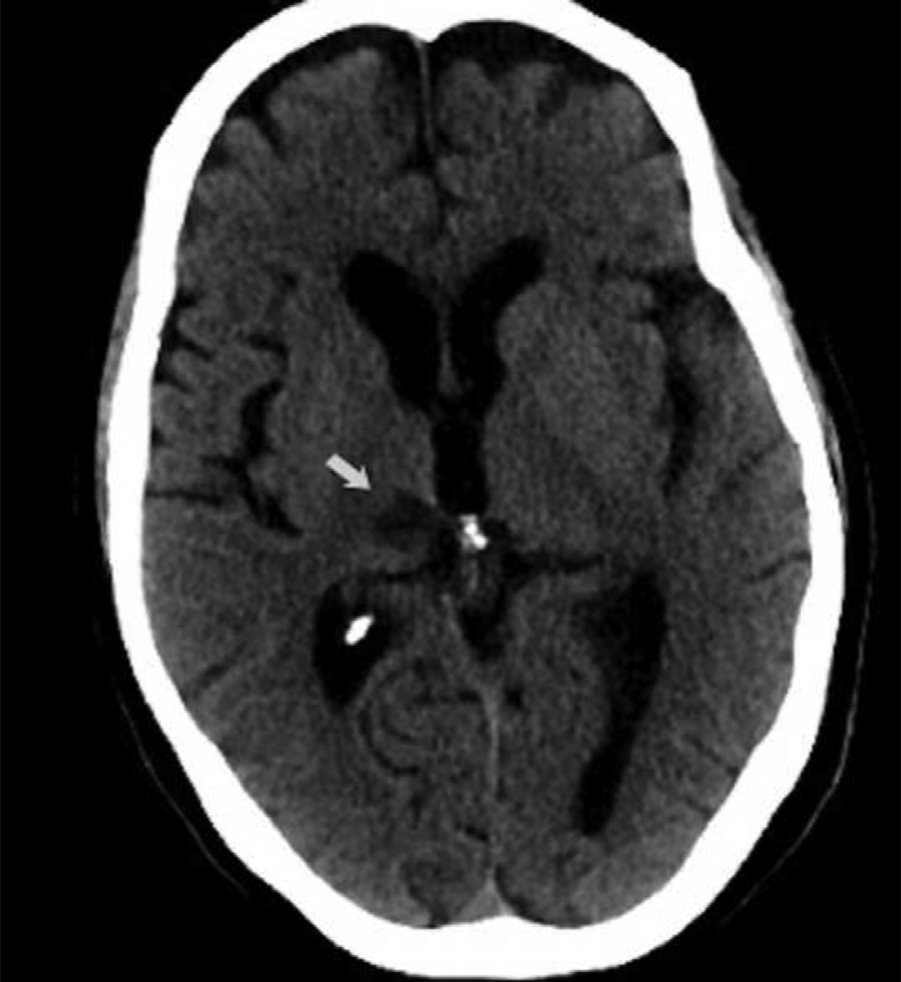
CT scan shows a hypodense area in the right thalamus.

**Fig. 2:**
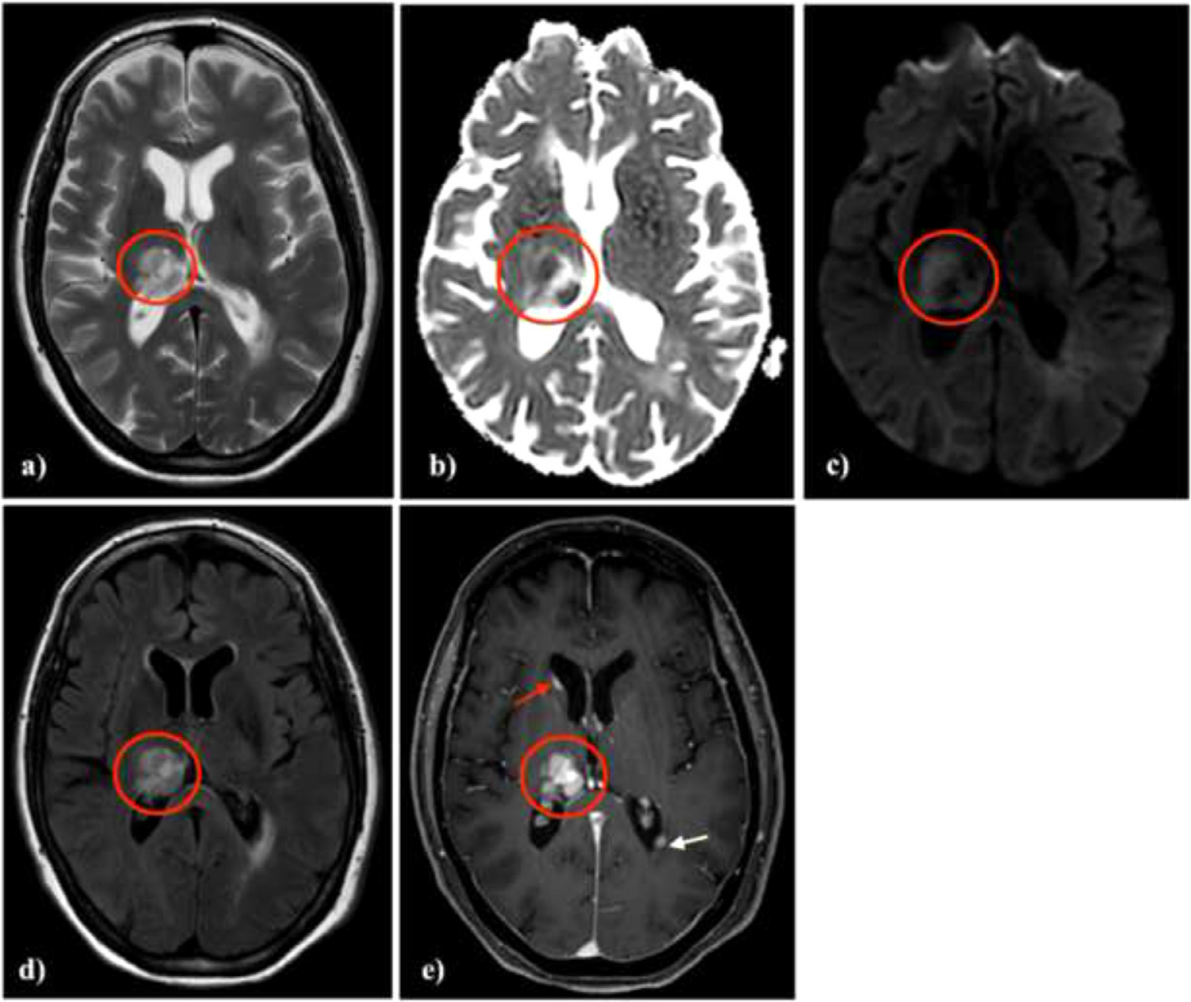
MR imaging: T2-weighted images demonstrate an inhomogeneous slightly hyperintense lesion in the right thalami, with little surrounding vasogenic edema (A, D) and restricted diffusion on DWI (B, C). Axial postcontrast T1-weighted image shows intense contrast enhancement of the thalamic lesion. Other two nodular enhancing lesions are localized in the left peritrigonal and periventricular white matter of the right frontal horn (E).

**Fig. 3:**
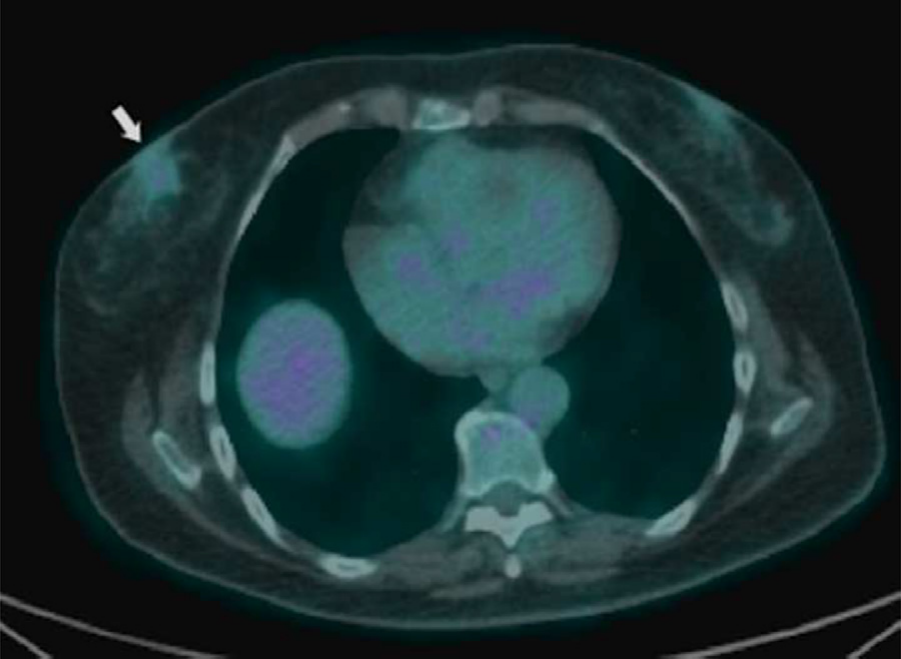
Right retroareolar lesion with mild FDG uptake on PET/CT (white arrow).

A previously healthy 56-year-old Caucasian woman attended the emergency department with a 1-month history of progressively worsening paraesthesia affecting the left upper extremity. The patient reported an episode of amaurosis and transient loss of consciousness. Emergency unenhanced head CT showed a hypodense area in the right thalamus (Fig. 1). Thereafter, the patient was hospitalized for further investigations considering the clinical condition and the imaging finding. During hospitalization, the patient underwent a brain magnetic resonance imaging (MRI) showing an inhomogeneous hyperintense lesion on T2-weighted images in the right thalamus demonstrating inhomogeneous reduction in the apparent diffusion coefficient with a median value of 0.6 × 10^−3^ mm^2^/s and intense enhancement after gadolinium administration (Fig. 2). Moreover, T1 postcontrast weighted images showed the presence of other 2 nodular enhancing lesions localized in the left peritrigonal and periventricular white matter of the right frontal horn (Fig. 2e). A relatively little vasogenic edema was associated with the tree lesions. A lumbar puncture was performed, and cerebrospinal fluid (CSF) analysis showed normal routine indices. CSF cytology was negative for neoplastic cells. Additionally, a positron emission tomography (PET/CT) scan was performed to exclude systemic disease. A nodular mass with mild metabolic activity (SUVmax 2.2) was detected in the retro-areolar region of the right breast, associated with nipple retraction and skin thickening (Fig. 3). Hypermetabolic right axillary lymphadenopathies were also observed.

**Fig. 4:**
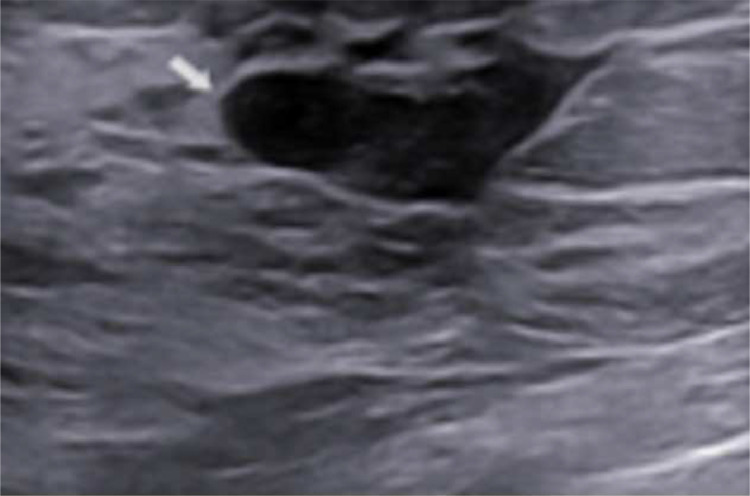
Right retroareolar hypoechoic lesion during tru-cut biopsy (white arrow).

A breast ultrasound was performed showing a hypoechoic mass with circumscribed and microlobulated margins (Fig. 4). Considering the need for an early diagnosis due to the brain findings and the clinical conditions of the patient, our expert breast center radiologists evaluated the need of an ultrasound-guided tru-cut biopsy of the breast lesion [13], and it was conducted immediately afterward (Fig. 4).

**Fig. 5:**
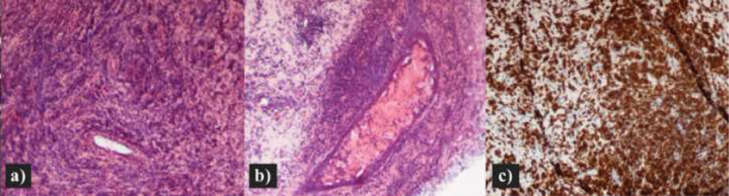
Breast biopsy: At low magnification (H&;E, 100×) a marked lymphocytic infiltration of the breast parenchyma with periductal disposition is observed (A, B). Small lymphocytes without obvious atypia are demonstrated showing diffuse membranous staining for CD3 (IHC, 100×) (C).

Histopathological examination revealed a diffuse parenchymal infiltration of small T lymphocytes with perivascular and periductal distribution and with no evident cytological atypia. At immunohistochemistry, the lymphocytes were diffusely CD3, CD5, and CD8 positive, with partial co-expression of PD-1. CD4, Granzyme B, CD56, CD20, TdT, and CD30 were negative. Ki-67 proliferation index was 5%. T-cell receptor (TCR) gene rearrangement testing revealed clonal TCRγ rearrangement (Fig. 5).

The findings were suggestive of an indolent CD8+ T-cell lymphoproliferative disease and appear to satisfy the diagnostic criteria for PBL.

**Fig. 6:**
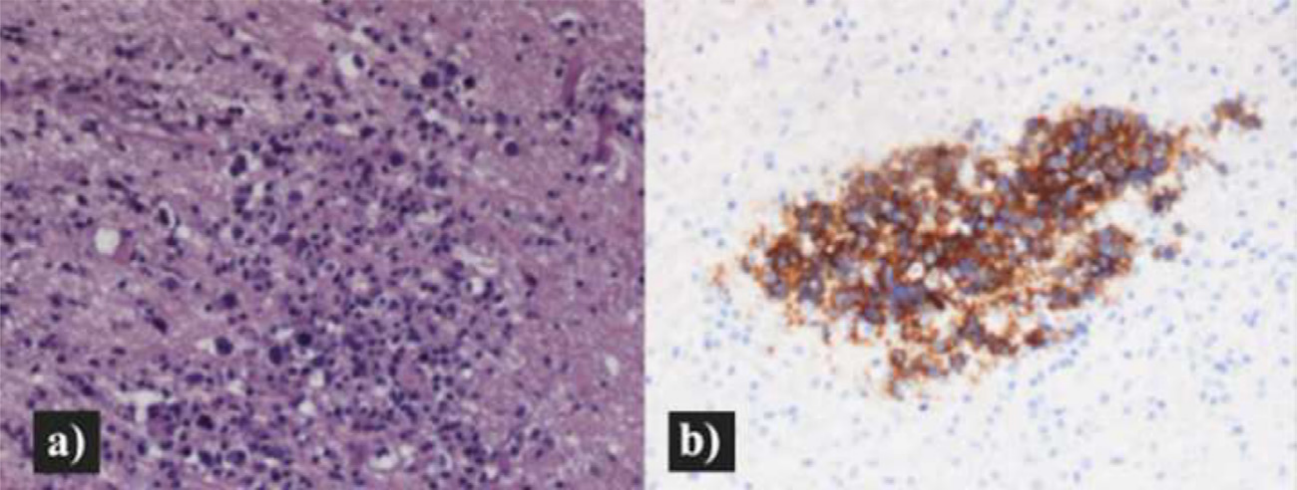
Brain histopathology: Infiltration of the brain tissue by large and atypical lymphoid cells (H&E, 200×) (A) with CD20 positivity (IHC, 200×) (B).

Meanwhile, also a stereotactic biopsy of the brain lesion was performed demonstrating diffuse perivascular infiltration of large atypical lymphoid cells. At immunohistochemistry, the atypical cells were CD20 and CD79a positive, CD3 and CD5 negative, with a Ki-67 proliferation index of 50%. Other stains revealed CD10 negativity, BCL2 positivity, BCL6 positivity in 60% of the cells, and MUM1 positivity in 50% of the cells (Fig. 6). These findings were consistent with DLCBL. A bone marrow biopsy was performed successively for staging purposes with no evidence of disease infiltration.

After histological diagnosis of PBL and PCNSL, the patient started chemotherapy with R-CHOP alongside with radiation therapy to chest wall and ipsilateral axilla.

**Fig. 7:**
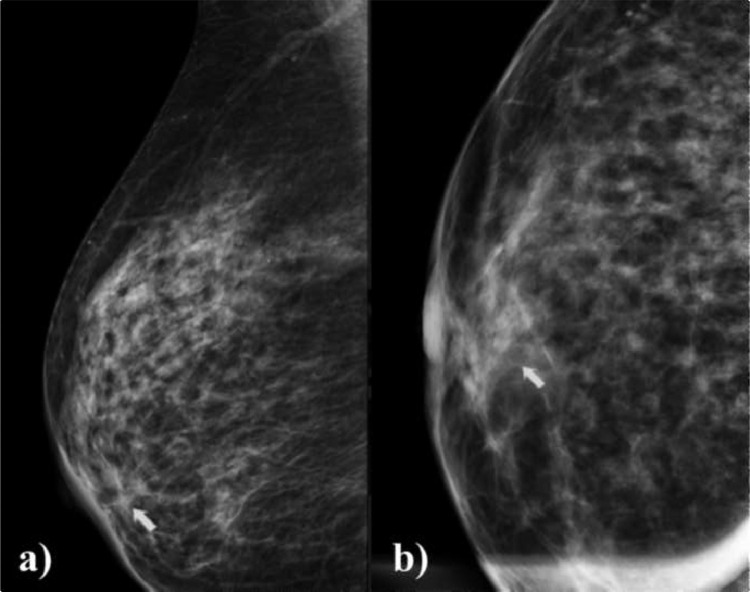
Mammographic imaging: Mediolateral oblique view (A) and compression magnification view in craniocaudal (B) revealed the presence of a retroareolar radio-opacity with indistinct margins and no calcifications (white arrow).

**Fig. 8:**
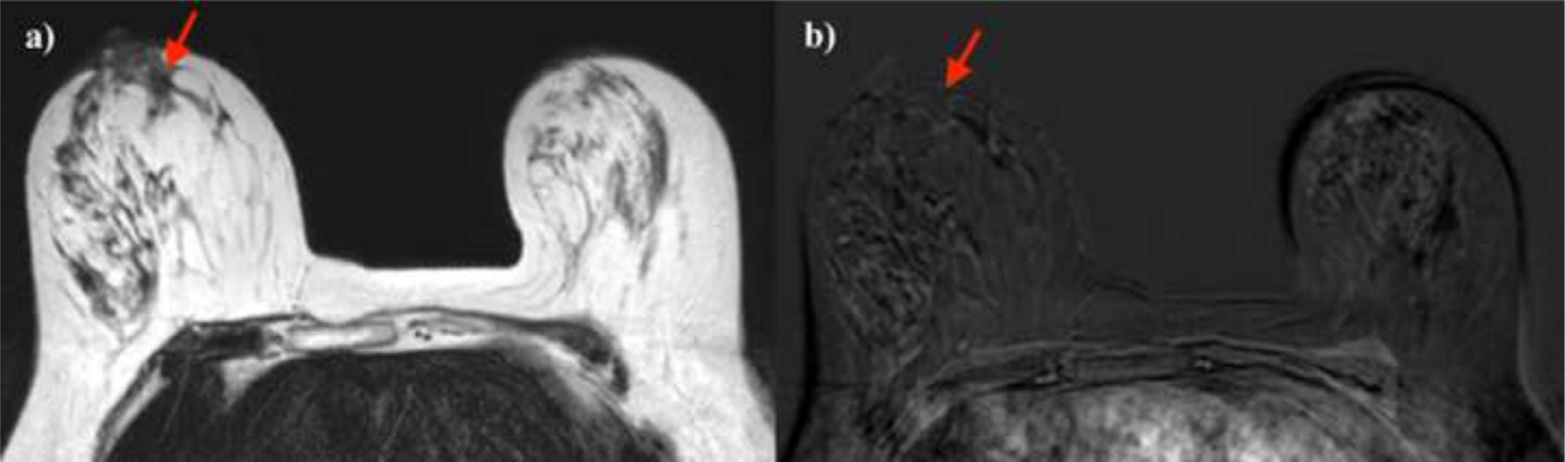
MR imaging after 21 days of treatment. T2-hypointense retroareolar area (A) with no significant contrast enhancement in fat-suppressed dynamic contrast-enhanced (DCE) images (B).

Breast MR imaging and mammography showed a good response to therapy after 21 days of treatment. Mammography revealed a retroareolar radio-opacity with indistinct margins and no calcifications (Fig. 7). MRI showed a T2 hypointense retroareolar mass with no contrast enhancement in fat-suppressed dynamic contrast-enhanced (DCE) images (Fig. 8).

As far as possible considering the absence of a pretreatment mammographic and MR imaging, the breast mass seemed approximately reduced in size compared to the PET/CT examination.

## Discussion

The presence of synchronous dual hematological malignancies is rarely reported in literature, even if it is thought to be an underestimate entity [14], PBL and PCNSL are 2 very rare types of NHL and to our knowledge this is the first case of PBL and PCNSL, with different histological subtypes, coexisting in an immunocompetent patient.

In our case, a DLCBL- PNCSL was diagnosticated representing the most common histological subtype according to literature, other rare subtypes include T-cell lymphoma, Burkitt's lymphoma, lymphoblastic lymphoma and marginal lymphoma [3,4]. PCNSL typically affects immunocompromised adults in association with Epstein-Barr virus (EBV), but it can also arise in immunocompetent patients who are usually elderly and EBV negative [2,15,16]. Several recent studies have shown an increase of PCNLS in immunocompetent patients, especially in the elderly [17,18]. Symptoms are extremely variable depending on the tumor location and size and may include altered mental status, focal neurological deficits, seizures, and increased intracranial pressure [19]. In immunocompetent patients PCNSL appears mostly as a solitary mass (66%), with a supratentorial location (86%) and often involving the periventricular white matter [20]. The most frequent locations of PCNSL are the cerebral hemispheres, followed by basal ganglia, thalamus, and corpus callosum [21]. Typical CT and MR imaging findings include a homogeneously enhancing parenchymal mass, with moderate peritumoral edema, generally less conspicuous than in metastases or high grade gliomas, probably due its infiltrative nature [22], [23], [24]. On precontrast MR images PCNSL usually appear hypo- or isointense on T1WI and variable on T2W and restricted diffusion on diffusion-weighted imaging. Calcifications, cystic changes, and hemorrhages can occur quite rarely and may be indicative of other diseases [23], [24], [25]. For the baseline staging, the International PCNSL Collaborative Group recommends brain and spinal cord magnetic resonance imaging, ophthalmologic and CSF evaluation plus PET/CT and bone marrow biopsy to detect involvement of other sites [26]. CFS analysis has proved to have an exceedingly low diagnostic capacity in immunocompetent patients with PCNLS [27].

Stereotactic brain biopsy is required to finally establish PCNSL diagnosis [26]. Current recommendations for the treatment of PCNLS consist of an induction poly-chemotherapy with high-dose methotrexate, an alkylating agent and rituximab (R-CHOP). If the patient has a good response to induction therapy, this is followed by consolidation therapy with either whole-brain radiotherapy or autologous stem-cell transplantation [28], [29], [30].

PBL account for less than 3% of extra nodal lymphomas and approximately 0.5% of breast malignancies [5], [6], [7], [8], [9], [10]. It typically affects women (98%) with an average age of presentation between 60 and 65 years old [5], [6], [8], [9], [30], [31], with a solitary and unilateral mass [7,32]. The most common histology is DLBCL, followed by follicular and mucosal-associated lymphoid tissue associated lymphomas. Breast involvement with T-cell lymphomas, as occurred in our case, is very rare, moreover it is almost exclusively reported in association with breast implants, in contradistinction to our case [5], [30], [31], [32], [33], [34], [35], [36], [37].

Clinically, the distinction between PBL and other breast tumors is often difficult, since both typically present with a painless breast mass [6] whereas constitutional symptoms are infrequent [6,32]. Cutaneous manifestations, nipple retraction and discharge are uncommon, usually associated with high-grade lymphoma or diffuse parenchymal involvement [10,38]. The imaging features are not specific making diagnosis extremely difficult. Screening mammography has less impact on the diagnosis of PBL than breast carcinoma [39]. At mammography PBL typically appears as a solitary mass, often with circumscribed margins, meanwhile spiculated images and architectural distortion are rarely identified. Infrequently PBL can be depicted only as structural asymmetries. Calcifications are usually absent [10,38,[40], [41], [42]]. US features of PBL are also nonspecific, depicted often as a hypoechoic or mixed echoic mass with circumscribed or indistinct margins, usually hypervascular on color Doppler sonography [10,38,40].

On MR imaging PBL appears as a round or oval mass characterized by hypointensity or isointensity at T1-weighted imaging and hyperintensity at T2-weighted imaging. Intense enhancement is usually demonstrated and shows most commonly a type II kinetic curve, with a plateau enhancement in the delayed phase, or more rarely a type III, with rapid enhancement and washout in the delayed phase [10,38,40,42].

Biopsy should be performed in order to establish a correct diagnosis, whereas PET/CT is recommended for evaluating metastatic disease and staging [38,40].

According to The International Extra-nodal Lymphoma Study Group, treatment management includes a combination of surgery, chemotherapy, and radiation depending on the type and stage of lymphoma [8,38].

## Conclusion

This is an unusual case of 2 rare forms of lymphoma coexisting in the same immunocompetent Patient without systemic involvement and history of hematological malignancy. To our knowledge, this is the first reported case of simultaneous DLBCL-PCNSL and T-cell PBL. Imaging findings are nonspecific for both PCNSL and PBL which can make early and accurate diagnosis extremely challenging with eventual consequence on the selection of the appropriate treatment strategies. This case report may open the way for a difficult and rare diagnosis.

## Patient consent

Informed consent was obtained from all individual participants included in the study. The participants have consented to the submission of the case report to the journal.
